# CD271^+^ stromal cells expand in arthritic synovium and exhibit a proinflammatory phenotype

**DOI:** 10.1186/s13075-016-0966-5

**Published:** 2016-03-15

**Authors:** Manuel J. Del Rey, Regina Faré, Alicia Usategui, Juan D. Cañete, Beatriz Bravo, María Galindo, Gabriel Criado, José L. Pablos

**Affiliations:** Servicio de Reumatología, Instituto de Investigación Hospital 12 de Octubre (i+12), 28041 Madrid, Spain; Unitat d’Artritis, Servei de Reumatologia, Hospital Clínic de Barcelona, Barcelona, Spain; Institut d’Investigacions Biomèdiques August Pí i Sunyer, Barcelona, Spain; Servicio de Cirugía Ortopédica y Traumatología, Hospital 12 de Octubre, Madrid, Spain

**Keywords:** Mesenchymal stem cells, Stromal cells, Synovial fibroblasts, Rheumatoid arthritis, Osteoarthritis, Nerve growth factor receptor

## Abstract

**Background:**

CD271^+^ stromal cells (SCs) with multipotent stem cell capacity have been identified in synovial tissues, but their functional significance is unknown. We analyzed the distribution of CD271^+^ cells in inflammatory synovial tissues as well as their ex vivo immunomodulatory and inflammatory phenotypes.

**Methods:**

CD271 expression was analyzed by immunohistochemistry in synovial tissues and by flow cytometry in primary adherent synovial cell cultures from rheumatoid arthritis (RA), osteoarthritis (OA), and non-inflammatory control tissues. Isolation of CD271^+^ synovial SCs was carried out by magnetic cell sorting. Allogeneic T-cell/SC cocultures were performed to analyze the regulatory capacity of these cells on T-cell proliferation and cytokine production. The production of inflammatory mediators was analyzed in cultures of sorted CD271^+^/^−^ SCs. The capacity of CD271^+^/^−^ SCs to induce inflammatory cell recruitment in vivo was evaluated in subcutaneous implants in immunodeficient mice.

**Results:**

CD271^+^ SC were detected in non-inflammatory as well as in arthritic synovial tissues with a specific perivascular distribution. CD271^+^ SC density was increased in RA and OA compared with normal synovial tissues. T-cell proliferation and cytokine synthesis were similarly modified by CD271^+^ and CD271^−^ SCs. Sorted CD271^+^ SCs from OA synovial tissues released significantly more interleukin (IL)-6, matrix metalloproteinase (MMP)-1, and MMP-3 than CD271^−^ SCs. In immunodeficient mice, implants of CD271^+^ SCs induced significantly higher myeloid cell infiltration than CD271^−^ SCs.

**Conclusions:**

Our results demonstrate that CD271^+^ perivascular SCs expand in RA and OA synovial tissues. CD271^+^ cells showed enhanced proinflammatory properties ex vivo and in vivo, whereas immunoregulatory properties were equivalent in CD271^+^ and CD271^−^ SC.

**Electronic supplementary material:**

The online version of this article (doi:10.1186/s13075-016-0966-5) contains supplementary material, which is available to authorized users.

## Background

The mesenchymal or stromal cell lineage in synovial tissue is represented by a heterogeneous population of stromal or fibroblastic cells, usually termed *synovial fibroblasts* (SFs) or *type B synoviocytes*. Under normal conditions, these cells contribute to the homeostasis of normal joints by synthesizing extracellular matrix components and secreting the specific components of synovial fluid [1]. SFs respond to inflammatory cytokines, notably tumor necrosis factor (TNF)-α, by producing a large variety of mediators of inflammation and tissue destruction [2, 3]. In chronic arthritis, SF hyperplasia and stable phenotypic changes also significantly contribute to the chronic inflammatory and destructive process [4–7].

SFs can be cultured as plastic adherent cells that express non-specific mesenchymal cell markers and lack myeloid and lymphoid lineage markers. However, SF cultures represent a heterogeneous cell mixture, as identified in synovial tissues by specific markers, and subpopulations are difficult to track under tissue culture conditions [4, 8–10]. Different synovial mesenchymal cell subtypes may have different differentiation and functional capacities that are difficult to analyze ex vivo.

Mesenchymal or stromal stem cells (MSCs) with multipotent differentiation potential and immunomodulatory activity have attracted strong interest as potential therapeutic agents [11, 12]. MSCs are abundant in the bone marrow (BM) as well as in most connective tissues, including synovial tissues [13, 14]. Because these cells lack specific markers, their identification in synovial tissues or SF cultures is difficult. CD271, also known as low-affinity nerve growth factor receptor (NGFR) or p75, identifies a subpopulation of stromal cells (SCs) with enhanced MSC differentiation capacities [15, 16]. Initially identified in the BM, CD271^+^ SC have subsequently been found in bone, adipose, and synovial tissues [16–18]. Their local roles in joint physiology or in the pathogenesis of arthritis as immunomodulatory cells are unknown. Different studies have shown their potential contribution to cartilage repair ex vivo [16, 17, 19], but it is not known whether CD271^+^ synovial SCs can play immunomodulatory functions in arthritic inflammatory processes.

We examined the distribution and abundance of CD271^+^ SCs in synovial tissues from patients with rheumatoid arthritis (RA) or osteoarthritis (OA), and from control subjects without inflammation, as well as their ex vivo immunomodulatory and proinflammatory capacities compared with CD271^−^ synovial adherent cells. We also analyzed their ability to induce inflammatory myeloid cell recruitment in vivo by engraftment in immunodeficient mice.

## Methods

### Ethical approval and consent to participate

This study was approved by the ethics committees of Hospital 12 de Octubre, Madrid, and Hospital Clìnic, Barcelona (Spain). All patients signed a written informed consent form. All animal experiments were carried out in accordance with institutional guidelines, and they were approved by the Hospital 12 de Octubre Animal Welfare Committee.

### Patients and synovial biopsies

Synovial tissues were obtained by arthroscopic biopsy of the knees of patients diagnosed with RA with active knee arthritis (*n* = 10) [20]. OA synovial tissues were obtained at prosthetic joint replacement surgery (*n* = 10). Normal non-inflammatory synovial tissues were obtained from individuals without joint disease at elective arthroscopy for minor traumatic lesions (*n* = 6).

### Immunolabeling

Synovial tissues were labeled by immunohistochemistry (IHC) using a standard peroxidase method (VECTASTAIN ABC Kit [standard]; Vector Laboratories, Burlingame, CA, USA) with anti-NGFR p75 (CD271) monoclonal antibody (mAb) (ME20.4 immunoglobulin G1 [IgG1]; Sigma-Aldrich, St. Louis, MO, USA) or mouse IgG1 isotype control (Sigma-Aldrich). Double-immunofluorescence (IF) labeling for CD271 and heat shock protein hsp47 (Assay Designs anti-hsp47 mAb M16.10A1 IgG2b; Enzo Life Sciences, Farmingdale, NY, USA) or CD31 (anti-CD31 rabbit mAb EPR3094 IgG; Abcam, Cambridge, UK) was performed using sequential incubation with isotype-specific Alexa Fluor 488 (green) or Alexa Fluor 594 (red) secondary antibodies (Molecular Probes/Life Technologies, Eugene, OR, USA). Counterstaining was done with hematoxylin or 4′,6-diamidino-2-phenylindole (DAPI) as indicated.

Frozen sections from mouse SC matrigel implants were also analyzed by IHC with an anti-human nuclei antibody (EMD Millipore, Temecula, CA, USA) or IF with phycoerythrin (PE)-labeled anti-CD11b (M1/70 IgG2b) or PE IgG2b control (BioLegend Inc., San Diego, CA, USA).

Photographs were obtained with Axioplan-2 fluorescence and LSM 510 META confocal microscopes (Carl Zeiss Microscopy, Jena, Germany). Quantitative data on CD271-stained fractional area and density of DAPI^+^ nuclei in matrigel sections were obtained from digitized images quantified using ImageJ software (http://rsb.info.nih.gov/ij; National Institutes of Health, Bethesda, MD, USA) as previously described [4].

### Synovial SC cultures

Primary (passage 0) synovial adherent SCs were obtained by explant growth of synovial tissue fragments from surgical OA tissues in Dulbecco’s modified Eagle’s medium/10 % fetal calf serum (FCS) (Lonza, Verviers, Belgium). CD271 expression was analyzed by flow cytometry (FC) with anti-CD271-allophycocyanin (APC) mAb (ME20.4 IgG1; Miltenyi Biotec, Bergisch Gladbach, Germany), and isolation of CD271^+^ and CD271^−^ cells was carried out by magnetic cell sorting using an LNGFR-APC MicroBead kit (Miltenyi Biotec). The purity of both populations was further analyzed by FC. Cells were replated, and supernatants were collected after 24 h for quantification of interleukin (IL)-6, IL-8, monocyte chemoattractant protein (MCP)-1, matrix metalloproteinase (MMP)-1, MMP-3, and vascular endothelial growth factor (VEGF) by multiplex enzyme-linked immunosorbent assay (ELISA) (RayBiotech, Norcross, GA, USA). To confirm the multipotent differentiation capacity of both CD271^+^ and CD271^−^ fractions, we performed subcultures under standard fat, bone, or cartilage differentiation conditions that confirmed their multipotential capacity (Additional file 1: Figure S1) [18]. IL-6 concentration was also determined by single, specific ELISA (BioLegend Inc.). SFs cultured at different passages under standard conditions from normal, OA, or RA tissues were also analyzed for CD271 expression by FC.

### Synovial SC/T-cell cocultures

Allogeneic T cells were isolated from peripheral blood from healthy donors by Ficoll-Hypaque density gradient centrifugation and negative selection using the Pan T Cell Isolation Kit II (Miltenyi Biotec). One day before coculture, CD271^+^ and CD271^−^ SCs (5 × 10^3^ cells/well) were seeded into 96-well plates in RPMI 1640/10 % FCS. Purified T cells (5 × 10^4^ cells/well) were labeled with CellVue Claret dye (Sigma-Aldrich) added to SC cultures and stimulated with human T-activator CD3/CD28 Dynabeads (1 bead/5 cells; Invitrogen Dynal, Oslo, Norway). After 24 h, supernatants were collected and IL-2, IL-10, IL-17, and interferon (IFN)-γ production was measured by ELISA (BioLegend Inc.). Proliferation was measured at 72 h by CellVue dye dilution as detected by FC.

### Mouse engraftment of CD271^+^ and CD271^−^ SCs

CD271^+^ or CD271^−^ SC implants were prepared by suspension of 7.5 × 10^4^ SCs, previously isolated as described above, in 250 μl of Matrigel growth factor reduced matrix (Corning Life Sciences, Bedford, MA, USA). The Matrigel/SC suspension was subcutaneously injected into the backs of NOD *scid* gamma (NOD.Cg-*Prkdc*^*scid*^*Il2rg*^*tm1Wjl*^/SzJ [NSG™]; The Jackson Laboratory, Bar Harbor, ME, USA) mice. Six independent experiments with CD271^+^/CD271^−^ SCs from six different OA membranes were performed. Mice were killed on the seventh day, and the skin containing Matrigel plugs was excised and snap-frozen in Tissue-Tek O.C.T. compound (Sakura Finetek, Alphen aan den Rijn, the Netherlands).

### Statistical analysis

Data were analyzed using GraphPad Prism v6.0 software (GraphPad Software, La Jolla, CA, USA). Means were compared by using Kruskal-Wallis, Friedman, or Wilcoxon tests as appropriate. A *p* value less than 0.05 was considered statistically significant.

## Results

### Expression of CD271^+^ in synovial tissues and cell cultures

**Fig. 1:**
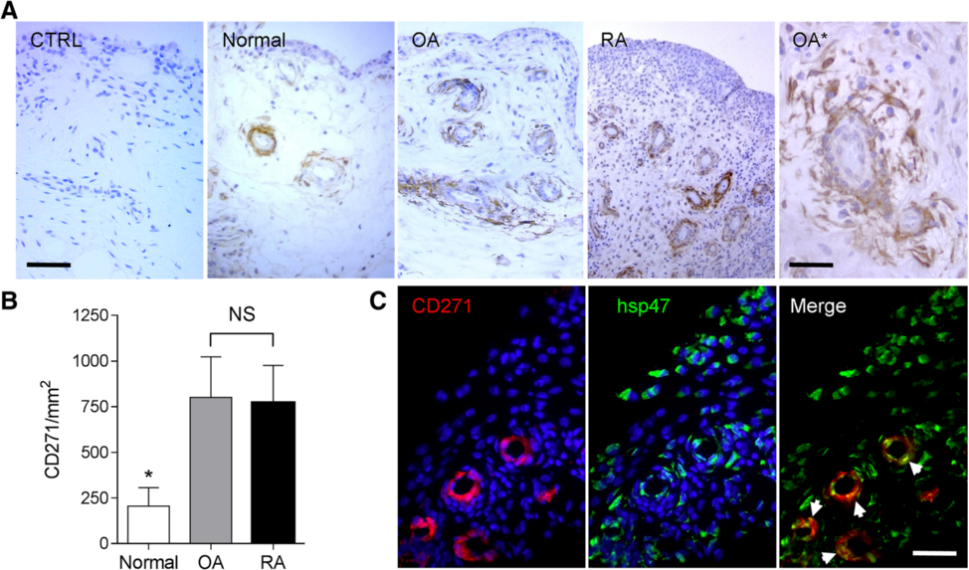
Expression of CD271 in synovial tissues. **a** Immunohistochemical immunoperoxidase labeling of CD271 in normal, osteoarthritis (OA) and rheumatoid arthritis (RA) synovial tissues as indicated (bar, 50 μm). *CTRL* immunoglobulin G1 isotype control. OA* shows a perivascular area in OA tissue with higher magnification (bar, 20 μm). **b** CD271 fractional immunostained area in the different groups (mean ± standard deviation). *p = 0.0012 by analysis of variance Kruskal-Wallis with Dunn’s multiple comparisons test. **c** Labeling of CD271 (*red*) and heat shock protein hsp47 (*green*). *Arrows* indicate double-positive CD271/hsp47 perivascular cells (bar, 20 μm). *NS* not significant

CD271^+^ cells were observed by IHC in all types of synovial tissues with a specific perivascular distribution, not restricted to pericytes but as an extended network around small and medium-sized blood vessels (Fig. 1a). The density of CD271^+^ cells was significantly increased in inflammatory tissues (OA and RA) compared with normal synovial tissues (Fig. 1b). To confirm the fibroblastic identity of synovial CD271^+^ cells, double-labeling for CD271 and the fibroblast marker hsp47 was performed [4]. We observed that CD271^+^ cells also expressed hsp47 in all cases, which confirmed that, in synovial tissues, CD271^+^ cells are fibroblastic cells (Fig. 1c). We also excluded endothelial identity of CD271 cells by double-labeling CD271 and CD31 (Additional file 2: Figure S2).

**Fig. 2:**
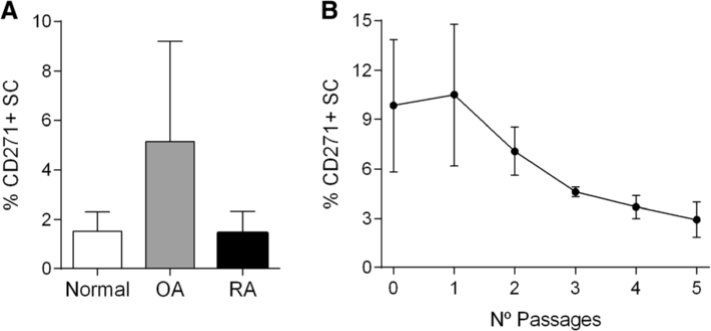
CD271^+^ cells in established synovial fluid (SF) cultures. **a** Percentage of CD271^+^ quantified by flow cytometry in passage 3 SF cultures from normal (*n* = 4), osteoarthritis (OA; *n* = 8), or rheumatoid arthritis (RA; *n* = 5) synovial biopsies. **b** Progressive reduction of CD271^+^ cells in SF cultures from OA synovial membranes (*n* = 3) throughout passages 0–5. *SC* stromal cells

To examine the presence of CD271^+^ cells in standard SF cultures, FC analysis of established (passage 3) SF cultures showed expression in a small but variable percentage of cells. A nonsignificant trend toward a higher proportion of CD271^+^ cells in SF from OA compared with RA or normal synovial tissues was observed (Fig. 2a). We also observed a progressive decrease in the percentage of CD271^+^ cells in individual SF cultures throughout passages 0–5 (Fig. 2b). Therefore, isolation of CD271^+^ SC for further studies was carried out in OA synovial cell cultures at passage 0. After magnetic cell sorting, purified cells contained 64 ± 12 % or 4.7 ± 4 % CD271^+^ cells in the CD271^+^ or CD271^−^ fraction, respectively (*n* = 19 sorting experiments).

### Immunomodulatory properties of CD271^+^/CD271^−^ SCs

**Fig. 3:**
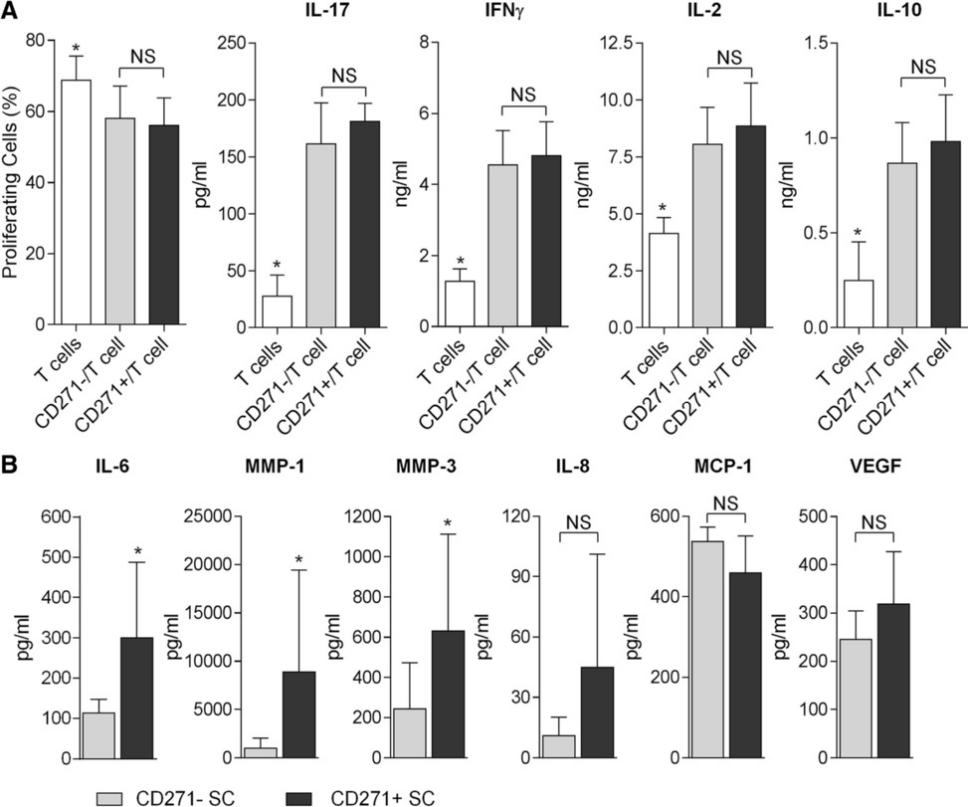
T-cell effects and synthesis of inflammatory factors by sorted CD271^+^ and CD271^−^ stromal cells (SC). **a** T-cell proliferation at 72 h and cytokine production at 24 h in supernatants of SC/T-cell cocultures stimulated with CD3/CD28 Dynabeads. Data are representative of paired CD271^+^ and CD271^−^ synovial SCs from nine different synovial tissue donors cocultured with T cells from nine allogeneic donors (mean ± standard error of the mean are indicated). *p < 0.05 by analysis of variance with Friedman and Dunn’s multiple comparisons tests. **b** Synthesis of inflammatory mediators in 24-h culture supernatants of paired CD271^+^ and CD271^−^ synovial SC cultures as quantified by multiplex enzyme-linked immunosorbent assay. Graphics represent triplicate samples from three patients with OA (mean ± standard deviation). *p < 0.05 by Wilcoxon test. *NS* not significant, *IL* interleukin, *IFN* interferon, *MMP* matrix metalloproteinase, *MCP-1* monocyte chemoattractant protein 1, *VEGF* vascular endothelial growth factor

To determine whether synovial CD271^+^ SCs differentially modulate T-cell effector functions, T cells were stimulated with anti-CD3/anti-CD28 beads in the presence of CD271^+^ and CD271^−^ cells (1:10 ratio of SCs to T cells). Under these conditions, both CD271^+^ and CD271^−^ SCs similarly inhibited the proliferation of allogeneic T cells (Fig. 3a).

Coculture of T cells with either CD271^+^ or CD271^−^ SC induced a similar increase in the production of IL-2, IL-17, IFN-γ, and IL-10 cytokines compared with T cells stimulated in the absence of SCs (Fig. 3a).

### Production of inflammatory factors by CD271^+^/CD271^−^ SCs

The production of selected proinflammatory mediators by SCs, including cytokines (IL-6, IL-8, MCP-1), metalloproteases (MMP-1, MMP-3), and angiogenic factors (VEGF), was quantified by multiplex ELISA in 24-h culture supernatants of sorted CD271^+^/CD271^−^ cells. CD271^+^ SCs released significantly more IL-6, MMP-1, and MMP-3 than CD271^−^ SCs did (Fig. 3b). A significant 3.1-fold increased production of IL-6 by CD271^+^ cell cultures was confirmed by single, specific ELISA in an extended set of samples (*n* = 5) (CD271^+^ SCs 1006 ± 286.4 pg/ml vs CD271^−^ SCs 320.2 ± 68.3 pg/ml [mean ± standard error of the mean], *p* < 0.05 by Wilcoxon test). No significant differences in the production of other mediators were observed (Fig. 3b).

### CD271^+^ SCs induce enhanced myeloid recruitment in vivo

**Fig. 4:**
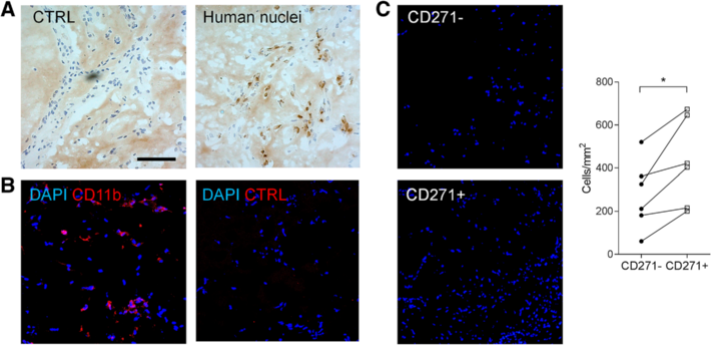
Analysis of CD271^+^ and CD271^−^ stromal cell (SC) implants in immunodeficient mice. **a** Engrafted SCs were identified in Matrigel implants by immunoperoxidase labeling with antihuman nuclei or isotype control (CTRL) (hematoxylin counterstaining; bar, 100 μm). **b** Identification on infiltrating myeloid cells by phycoerythrin (PE)-CD11b antibody (*red*) and 4′,6-diamidino-2-phenylindole (DAPI) counterstaining (*blue*), PE–immunoglobulin G2b isotype control is also shown. **c** Representative images of sections stained with DAPI from CD271^−^ and CD271^+^ Matrigel implants (*left panel*). Quantitative analysis of cellularity (DAPI-positive nuclei per square millimeter) in CD271^−^ and CD271^+^ implants (*right panel*). Data are from six different OA synovial membranes, sorted as CD271^+^ or CD271^−^ SCs and implanted into NSG mice. *p = 0.03 by Wilcoxon test

To examine the proinflammatory properties of CD271^+^ SCs in vivo, we implanted isolated CD271^−^ or CD271^+^ cells in a Matrigel matrix by subcutaneous injection into NSG mice. After 7 days, human SCs persisted in the implants, as demonstrated by antihuman nuclei IHC, and induced abundant cell recruitment into the Matrigel implant. These infiltrating cells were identified as CD11b^+^ mouse myeloid cells (Fig. 4a and b).

The cellularity of the infiltrate was highly variable in implants containing SCs from different individuals. However, in a paired analysis, infiltrating cells were significantly increased in the implants of mice engrafted with CD271^+^ SCs compared with those in mice engrafted with CD271^−^ SCs from the same individual (Fig. 4c).

## Discussion

CD271^+^ SCs were first described in the BM and considered as a network of specialized perivascular cells [15]. Sorted BM CD271^+^ SCs have shown multilineage differentiation and immunomodulatory properties as unselected BM MSCs [21]. CD271^+^ SCs have also been described in inflammatory synovial tissues, where a potential role in cartilage repair has been proposed [16, 17, 19]. Our data confirm that, in synovial tissues, a similar perivascular network of CD271^+^ is present, similar to that previously observed in the BM, and this prompted us to study their potential function in arthritis.

Because BM CD271^+^ cells have been shown to display ex vivo immunomodulatory properties similar to those of MSCs, we analyzed whether synovial CD271^+^ cells may also play this function. Our findings demonstrate that both CD271^+^ and CD271^−^ synovial SCs have similar antiproliferative effects on allogeneic stimulated T cells. Although consistent with previous studies on CD271^+^ cells, this suggests that this property is not specific to MSCs, because CD271^−^ synovial SCs also serve this immunomodulatory function, consistent with previous studies with other types of human differentiated fibroblasts [22]. We also found a potent, nonspecific stimulatory effect on T-cell cytokine synthesis in both CD271^+^ and CD271^−^ synovial SCs. This effect was not selective and involved IL-2 and both regulatory (IL-10) and effector (IL-17 and IFN-γ) cytokines. Therefore, although these data confirm MSC-like immunomodulatory properties of synovial CD271^+^ cells, they are not distinctive of these cells, reinforcing the concept that these properties could be shared by different stromal or fibroblastic cell types [22].

Expansion and phenotypic changes of differentiated SFs characterize chronic arthritic conditions [4]. We also found significant hyperplasia of perivascular CD271^+^ SCs in both OA and RA inflammatory tissues compared with noninflammatory tissues, which is consistent with previous observations in OA [19]. Interestingly, these cells show an enhanced intrinsic capacity to synthesize the proinflammatory cytokines IL-6 and MMPs ex vivo, suggesting that they could play a pathogenic rather than a protective role in arthritis. Indeed, we confirmed an intrinsically enhanced proinflammatory function of CD271^+^ SCs resulting in increased myeloid cell infiltration in the absence of additional stimuli in immunodeficient mice. Although analyzed chemokines were not significantly overexpressed by cultured CD271^+^ cells, myeloid cell recruitment could be secondary to in vivo IL-6 effects such as endothelial activation and induction of chemokine expression [23, 24].

In agreement with this interpretation, a CD271^+^ perivascular fibroblastic pool present in normal mouse tissues has been shown to expand and contribute to inflammation and scarring upon tissue injury [25]. Therefore, CD271^+^ might identify a reservoir of perivascular SCs with enhanced inflammatory mediator synthesis that expands in arthritis. However, due to limited CD271^+^ cell availability from RA tissues, we analyzed only cultured OA cells and therefore potential differences between OA and RA CD271^+^ SC cannot be discarded.

Systemic intravenous administration of allogeneic or xenogeneic MSCs has been shown to ameliorate immunomediated inflammation in different experimental models, including collagen-induced arthritis in mice [26]. Whether such an effect is mediated by their homing into the inflamed tissues is unclear [27]. So far, no studies have assessed whether synovial homing contributes to the effect of systemic infusion of different types of SCs, including SF in arthritis. Our data do not support a local protective or anti-inflammatory role for CD271^+^ SCs. The observed expansion in OA and RA synovial tissues might rather contribute to inflammatory mediator synthesis and myeloid cell infiltration. This suggests that different types of synovial SCs, including CD271^+^ cells or differentiated pathogenic SF, may have overlapping functions in arthritis. Improved knowledge of the relationship between different synovial SC populations is needed to understand their participation in arthritic diseases.

## Conclusions

CD271^+^ SCs with a perivascular distribution are present in all synovial tissues and expand in inflammatory conditions (RA and OA). CD271^+^ and CD271^−^ SCs have similar immunomodulatory properties ex vivo, whereas CD271^+^ cells show enhanced proinflammatory features ex vivo and in vivo. These observations provide a better understanding of the diverse contributions of different synovial SC subpopulations in arthritic diseases.

## Additional files

Additional file 1: Figure S1. Multipotent differentiation capacity of SCs. CD271^+^ and CD271^−^ synovial SCs were cultured under specific adipocytic, chondroblastic, or osteoblastic differentiation conditions. Representative images of differentiated SC cultures stained with oil red for adipocytes (**a**), Alcian Blue for chondroblasts (**b**), and Alizarin Red for osteoblasts (**c**). Original magnification, ×100. (PDF 258 kb) Additional file 2: Figure S2. Double CD31/CD271 labeling of synovial tissues. Representative images of synovial tissues labeled for CD271 (*red*), CD31 (*green*), and DAPI counterstaining (*blue*) showing small vessels (**a**), medium-size vessels (**b**), and isotype control (**c**). Original magnification, ×400; bar, 44 μm. (PDF 167 kb)

## Abbreviations

APC: allophycocyanin
BM: bone marrow
DAPI: 4′,6-diamidino-2-phenylindole
ELISA: enzyme-linked immunosorbent assay
FC: flow cytometry
FCS: fetal calf serum
hsp: heat shock protein
IF: immunofluorescence
IFN-γ: interferon-γ
IgG: immunoglobulin G
IHC: immunohistochemistry
IL: interleukin
mAb: monoclonal antibody
MCP-1: monocyte chemoattractant protein 1
MMP: matrix metalloproteinase
MSC: mesenchymal or stromal stem cell
NGFR: nerve growth factor receptor
NS: not significant
OA: osteoarthritis
PE: phycoerythrin
RA: rheumatoid arthritis
SC: stromal cell
SF: synovial fibroblast
TNF-α: tumor necrosis factor-α
VEGF: vascular endothelial growth factor
