# Supplementary figures and images for: CD271+ stromal cells expand in arthritic synovium and exhibit a proinflammatory phenotype
Source: Arthritis Res Ther. 2016 Mar 15;18:66. doi: 10.1186/s13075-016-0966-5 (PMC4791981; doi:10.1186/s13075-016-0966-5)

**CD271+**

**CD271-**

**A**

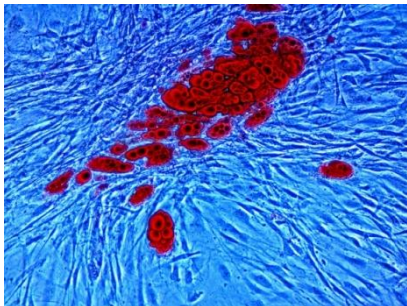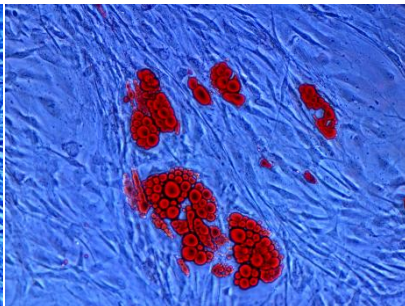

**B**

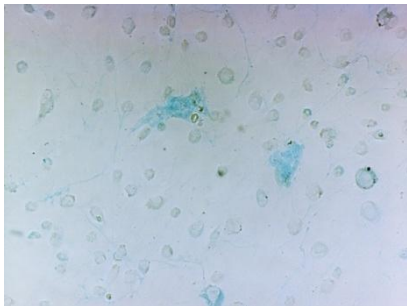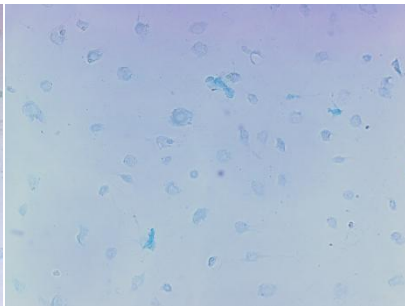

**C**

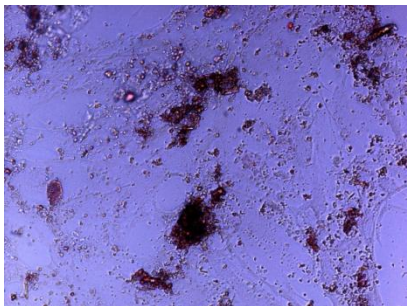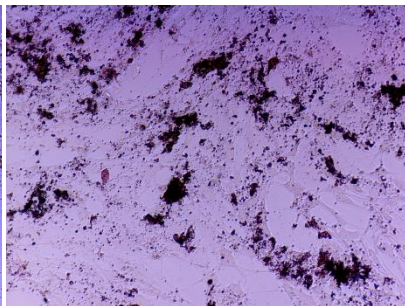

Supplement: Additional file 1: Figure S1. — Multipotent differentiation capacity of SCs. CD271+ and CD271− synovial SCs were cultured under specific adipocytic, chondroblastic, or osteoblastic differentiation conditions. Representative images of differentiated SC cultures stained with oil red for adipocytes (a), Alcian Blue for chondroblasts (b), and Alizarin Red for osteoblasts (c). Original magnification, ×100. (PDF 258 kb) [file 13075_2016_966_MOESM1_ESM.pdf]

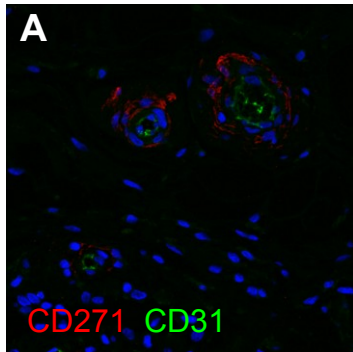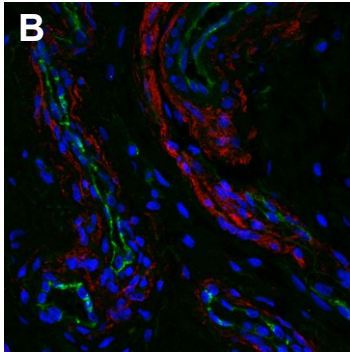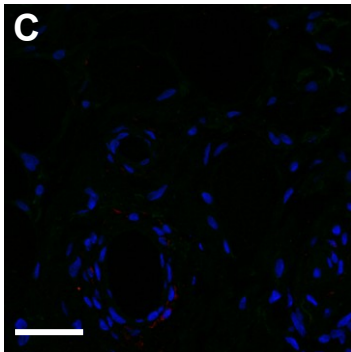

Supplement: Additional file 2: Figure S2. — Double CD31/CD271 labeling of synovial tissues. Representative images of synovial tissues labeled for CD271 (red), CD31 (green), and DAPI counterstaining (blue) showing small vessels (a), medium-size vessels (b), and isotype control (c). Original magnification, ×400; bar, 44 μm. (PDF 167 kb) [file 13075_2016_966_MOESM2_ESM.pdf]
